# Nutrition Claims Frequency and Compliance in a Food Sample of the Spanish Market: The BADALI Study

**DOI:** 10.3390/nu12102943

**Published:** 2020-09-25

**Authors:** Ana B. Ropero, Nuria Blain, Marta Beltrá

**Affiliations:** Institute of Bioengineering, Miguel Hernández University, 03202 Elche, Alicante, Spain; nuriablain@hotmail.com (N.B.); beltra@umh.es (M.B.)

**Keywords:** nutrition claims, regulation compliance, food labelling, consumer, public health

## Abstract

Nutrition claims (NCs) have been shown to affect customers’ perceptions and behaviour. In Europe, they are regulated by Regulation (EC) No 1924/2006. The aim of this work was to analyse the prevalence and compliance of NCs according to this regulation in Spain. For this purpose, we used the BADALI database, which included 3197 foods present in the Spanish market. Our results show that 36.1% of all foods carried NCs, at a rate of 3.3 NCs/food. The prevalence was very heterogeneous among food groups. Nuts and seeds, legumes and non-alcoholic beverages were the groups with the highest prevalence. Micronutrients, fat, fibre and sugars were the nutrients most referred to in NCs. Overall, the compliance was low, with 49.2% NCs correct. Fibre and proteins were the nutrients with most correct NCs. Vegetables and non-alcoholic beverages were the food groups with the highest proportion of correct NCs. The main reason for incorrect NCs was because the amount of the nutrient was not stated in the label. The results of our study reveal that the aim of the European Commission to ensure a high level of protection for consumers regarding NCs has not been fulfilled. Therefore, we consider it crucial that European institutions invest in guaranteeing regulation compliance.

## 1. Introduction

The European Commission (EC) defines “claim” as “any message or representation, which is not mandatory under Community or national legislation, including pictorial, graphic or symbolic representation, in any form, which states, suggests or implies that a food has particular characteristics” [1]. “Nutrition claim means any claim which states, suggests or implies that a food has particular beneficial nutritional properties due to: (a) the energy (calorific value) it (i) provides; (ii) provides at a reduced or increased rate; or (iii) does not provide; and/or (b) the nutrients or other substances it (i) contains; (ii) contains in reduced or increased proportions; or (iii) does not contain” [1]. According to Article 8.1, “Nutrition claims shall only be permitted if they are listed in the Annex and are in conformity with the conditions set out in this Regulation” [1].

Regulation (EC) No 1924/2006 was the result of the growing concern about the unregulated use of nutrition and health claims over recent decades to promote product selling. In fact, EC stated in 2006 that “An increasing number of foods labelled and advertised in the Community bear nutrition and health claims. In order to ensure a high level of protection for consumers and to facilitate their choice, products put on the market, including imported products, should be safe and adequately labelled” [1]. The governments of other countries, such as Australia-New Zealand, USA and Canada, have issued similar regulations [2,3,4].

The EC considers that nutrition and health claims confer a positive image to the foods bearing them [1]. In fact, some authors talk about a ‘health halo’ effect, “whereby an individual generalises from a nutrition or health claim that a product is healthier or has more favourable attributes than it actually does” [5,6,7,8]. Some reviews suggest that nutrition claims (NCs) can actually influence food choices [9,10,11]. Recent systematic reviews indicate that products bearing a NC have more probabilities to be chosen and that they can alter adults’ perceptions concerning the content of the products [9,12]. Another systematic review concluded that NCs related to fat, sugar and energy content can influence the perceived healthfulness of products, making them seem healthier than they are, and thus influencing food purchase intentions [10]. Some factors related to customers may also be important to the effect of NCs. Nutrition knowledge, motivation to adopt a healthy diet, health consciousness and consumers’ difficulty to understand claims may modulate the effect of NCs [9,10,13,14,15,16]. As a summary of all the potential factors influencing the effect of NCs on food choices, Oostenbach proposed a conceptual model in 2019 [10].

Proper food choices are essential in following a healthy diet. According to the Global Burden of Diseases, Injuries and Risk Factors Study (GBD), dietary risks are responsible for more than 10 million deaths in the world [17]. Unhealthy diets increase the risk of dying from a noncommunicable disease (NCDs), along with tobacco use, physical inactivity and the harmful use of alcohol [18].

Given the influence of NCs in several aspects related to food intake and after more than 10 years of implantation, we considered it important to review the European Regulation (EC) No 1924/2006 compliance in Spain. Therefore, we aimed to study the prevalence and compliance of NCs in a sample of foods sold in the Spanish market.

## 2. Materials and Methods

### 2.1. BADALI Database of Food Products Available in the Spanish Market

The data used in this work come from the BADALI database. This database was initially developed as a social project to improve citizen’s diets and can be freely accessed online in the BADALI webpage [19]. Food information included in the database and used in this study was collected from August 2013 to April 2019 from brands’ webpages (total of 159 different brands). In a first phase, brands were chosen according to information available in their web pages following no specific pattern. No retailer brands were collected. The inclusion criteria for brands were: (1) they belonged to companies located in Spain, according to the information provided in the webpage, or (2) the “Spain” version of the webpage was available for international brands. The inclusion criteria for foods were: (1) to be sold in the Spanish market and (2) to have the nutrient declaration on the webpage. Foods from different groups were collected in order to provide consumers with variety. Since most foods were considered unhealthy following the internal criteria [20], brands with healthier foods were subsequently chosen in a second phase, to provide consumers with healthier alternatives. Fresh foods were very poorly represented in the database because they are exempt from the mandatory nutrition declaration, according to annex V of Regulation (EC) No 1169/2011 [21]. Nutrient composition data were reviewed by the authors and inconsistent information was not used for further analysis. Foods were classified into groups following the Spanish Food Code [22] (Table S1). Further classification into subgroups was performed in order to reduce heterogeneity and to analyse specific types of foods (Table S1).

### 2.2. Nutrition Claims Classification and Compliance

NCs were collected from brand webpages. Only those displayed as text were considered for analysis, including text in the images of packages. NCs displayed as pictorial, graphic or symbolic representations were uncommon and could easily be misinterpreted. Therefore, they were not searched for. Article 8 of Regulation (EC) No 1924/2006 states, “Nutrition claims shall only be permitted if they are listed in the Annex and are in conformity with the conditions set out in this Regulation” [1]. NCs not included in the annex, either specific or general (such as “nutritious”), were considered non-authorised and classified as incorrect. Therefore, the category “non-authorised” was used for these NCs. Claims regarding specific ingredients or products, meals or dishes prepared with the actual food were not considered NCs of the foods and, therefore, were not analysed. Authorised NCs were grouped into categories for easier analyses, according to the food component claimed (energy or nutrient). Comparative NCs followed this classification, except when the nutrient referred to was not included in any category (cholesterol, carbohydrates and starch). In such cases, a new category was stablished (“reduced (other)”).

To determine NCs’ compliance, both Regulation (EC) No 1924/2006 and the “Guidance on the implementation of Regulation No 1924/2006” were followed [1,23]. In addition, the guidance of the Spanish Agency for Food Safety and Nutrition (AESAN) was used when required [24]. By the time food information was collected in the database, the regulation was already in force, except for specific products bearing trademarks or brand names (not applicable to foods in the database used) [1]. When ingredients were required to evaluate compliance with any NC, only the information provided in the brand webpage was considered (consulted at any moment). Some of the NCs could not be evaluated because: (1) ingredients were required for the evaluation, but they were not available in the brand’s webpage; (2) there were possible errors in the amount of the nutrient/energy claimed; (3) other reasons, such as energy not being provided although it was required to make the appropriate calculations, or the absence of reference foods to compare to. Therefore, they were classified as “non-evaluable” when compliance was analysed.

NCs about saturated fat were slightly more complex to evaluate than the rest because trans fatty acids content in the product had to be considered in order to use these claims [1]. According to Article 7 of Regulation (EC) No 1924/2006, the amount of the nutrient referred to in the NC has to be provided by the manufacturer [1]. Therefore, the absence of trans fatty acids content in a product with an NC about saturated fat qualifies as a reason for failing to comply with regulation. None of the products bearing an NC displayed trans fatty acids content. However, we only ascribed incorrect NCs to trans fatty acids when the following conditions related to saturated fats were met, i.e.,:

- The amount of saturated fats was provided by the manufacturer;
- Saturated fat content by itself did not exceed the maximum for both saturated fat and trans fatty acids stablished by the annex of Regulation (EC) No 1924/2006 [1];
- Comparative NCs could be correct if only saturated fat were considered.

Article 9 of Regulation (EC) No 1924/2006 states that comparative claims “may only be made between foods of the same category, taking into consideration a range of foods of that category” [1]. However, to facilitate the analysis, reference products from the Spanish Food Composition Database, (BEDCA) were used when available [25]. Whenever this was not possible, similar products from different brands included in the BADALI database were used. Finally, the original product of the same brand was used whenever the other two options were not available.

When required, some extra criteria for the analysis of NCs compliance had to be stablished:

- “Rich in”—There is a contradiction between Regulation (EC) No 1924/2006 and the interpretative note by AESAN. The first indicates that the conditions for “high” are to be used, while “source of” is suggested by AESAN [1,24]. The criteria in the regulation were followed;
- The NC “naturally/natural” was evaluated individually. To be considered correct, the associated NC had also to be correct;
- Some NCs were of the type “with/high minerals”, “with/high vitamins” or “with vitamin B”. These were only considered correct when the equivalent NCs for at least two individual minerals/vitamins were correct. In this case, only individual NCs were taken into consideration for statistics. Otherwise, the general NC was considered incorrect. If only the NC for one individual mineral or vitamin was correct, this was considered so, in addition to the general incorrect claim;
- In the case of claims of the kind “full of vitamins/minerals” or “the food contains many vitamins/minerals”, the procedure mentioned above was followed, with a minimum of three vitamins/minerals required to be considered correct;
- Claims of the kind “source of vitamins (B, B9, B5, B6, E)” were considered as four individual NCs (B9, B5, B6, E);
- A claim of the kind “7 vitamins” was considered as seven incorrect NCs if it was so for any vitamin. If it was correct for a number of vitamins, it was considered so for those vitamins, while incorrect for the rest;
- Some wording flexibility rules were required:
  ○ Excellent/Large source of = high;
  ○ (Very) few calories = low energy;
  ○ Important source of = high;
  ○ A small part of (sugar) = low (sugar);
  ○ With very little fat = low fat;
  ○ Full of = high;
  ○ Very rich = high;
  ○ Mentioning a nutrient was interpreted as “source of” that nutrient.
- Comparative claims were considered incorrect when they were made about foods of different categories [1] (article 9);
- NCs such as “0%”, “0” or “zero” with no indication of the nutrient/energy they refer to were considered incorrect;
- When required, rounding guidelines were used, following the guidance document by the European Commission [26].

## 3. Results

### 3.1. Food Population Description and General Nutrition Claims Prevalence

**Figure 1:**
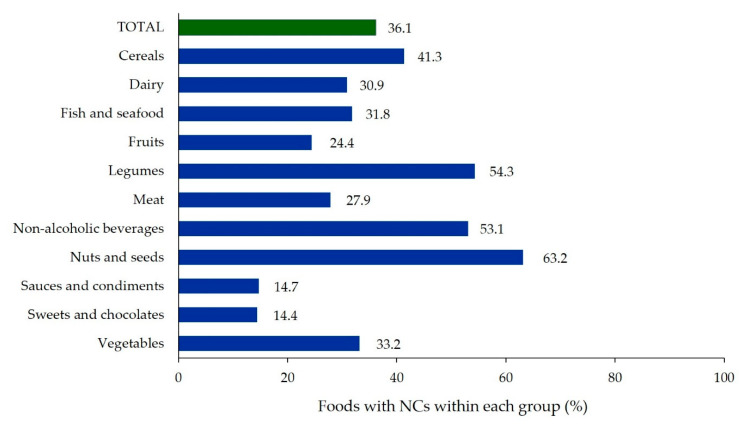
Foods with NCs by group (%). (Note: percentage was calculated using data in Table 1).

A total of 3197 foods were analysed for NCs. The most represented food group was that of cereals, followed by dairy and fish and seafood (Table 1). The fat group was the least represented. Because of the small sample size, fats were not analysed as an independent group in Figure 1 and Table 2 and Table S2. Among the subgroups, processed fish and seafood, chocolates and biscuits had more than 200 foods each (Table S2).

As shown in Table 1, 36.1% of all foods analysed carried NCs. Cereals was the group with more foods with NCs, followed by legumes, non-alcoholic beverages and dairy. When comparing individual groups, as many as 63.2% of nuts and seeds carried NCs, while legumes and non-alcoholic beverages also surpassed 50% (Figure 1). Specifically, all foods in the legumes/flour and pasta-like subgroup bore NCs, while the legumes/other derivatives and processed, other beverages and breakfast cereals and flakes had more than 70% prevalence (Table S2).

A total of 3839 NCs were identified (Table 2), at a rate of 3.3 NCs/food (considering only those bearing NCs). The distribution of these claims was heterogeneous, with 33% of foods bearing only one NC and 75.9% carrying 1–4 NCs (Figure S1). It is remarkable that 214 foods carried more than five NCs and 45 more than 10 (29 were nuts and seeds, and 10 cereals).

The group contributing most to the number of NCs was cereals, with more than 1000 (Table 2). Legumes, nuts and seeds and non-alcoholic beverages followed, with more than 500 each. In addition, legumes and nuts and seeds had the highest rate of NCs per food. As for subgroups, seven presented more than 200 NCs/each (legumes/other derivatives and processed, biscuits, breakfast cereals and flakes, natural or toasted, pasta and rice, other beverages and the two subgroups of nuts and seeds) (Table S2). Due to the small number of NCs, the sauces and condiments group was not analysed individually.

### 3.2. Nutrition Claims by Nutrients and Food Group

Authorised NCs represented 90.8% of all the claims and 8% were comparative claims. Non-authorised claims were also quite abundant, 9.2%, considering that they should not have been used.

Table 2 and Table S3 show all the data regarding NCs by nutrient and food group. Fats and vitamins were the nutrients most frequently claimed, followed by minerals and fibre (Table 2). The most frequent specific NCs were “source of fibre”, “sugars-free”, “high fibre”, “low fat” and “low saturated fat” (Table S3). “High protein”, “with no added sugars”, “source of protein” and “source of calcium” also surpassed 100 NCs (Table S3). The most frequent comparative claim was “light/lite” and those about salt. As for non-authorised NCs, they were distributed among general claims or those regarding specific nutrients (Table S4).

When NCs were analysed by nutrient and group, the distribution was quite heterogeneous. As shown in Table 2, together, vitamins and minerals were the most claimed nutrients for five food groups (cereals, dairy, fruits, non-alcoholic beverages and nuts and seeds). Micronutrients received more than half of all the NCs for nuts and seeds (286 of 557) (Table 2), with phosphorus, magnesium, iron and vitamin E as the most frequent (39–42 NCs/each). As expected, calcium was the predominant mineral for dairy (44 of 50) and legumes (34 of 85). Curiously enough, iron was the subject of only a few NCs in legumes (17 of 85), but it was the most claimed mineral for cereals (37 of 100). Vitamins B received more than half of all the NCs about vitamins in the cereal group (137 of 225).

Almost half of all the NCs about fibre were on cereals (47.4%) (Table 2), with biscuits as the subgroup with the highest prevalence of all (76 of 454 total NCs about fibre). Fibre was also the main nutrient claimed for vegetables (Table 2), mostly “high fibre” (49 of 55) and predominantly in canned vegetables (44 of 49).

As shown in Table 2, NCs about fat were very frequent for dairy, fish and seafood, legumes and meat. In fact, 50.8% of all NCs on meat were about fat, mostly “low fat” (36 of 61). Omega-3 was the most frequently claimed fat for fish and seafood (56 of 83). Fermented soy and soy desserts accounted for 66% of all NCs about fat in the legumes group, with a rate of more than two claims/food. As for dairy, all the NCs about fat in the fermented milk subgroup were fat-free (28).

Cereals took up as much as 40.6% of all the claims about sugar (Table 2). However, it is also important to mention that claims about this nutrient represented 60% of all the claims in sweets and chocolates (Table 2), mostly on chocolates (39 of 42). Sugar was also the most claimed nutrient for fruits (Table 2), with 25 of the 31 in jam.

Nearly half of the NCs about energy were on non-alcoholic beverages (Table 2), mostly on fruit beverages and soft drinks (38 of 43 NCs about energy in non-alcoholic beverages). Legumes accounted for one third of all the NCs about proteins (Table 2) and half of them were made on other derivatives and processed (47 of 92). Most of the claims “Naturally/natural” were made on fish and seafood and legumes (Table 2). As for non-authorised NCs, cereals and legumes were the most predominant groups (Table 2).

### 3.3. Regulation Compliance

Surprisingly, less than half of the NCs were correct according to Regulation (EC) No 1924/2006 [1] (Table 2). Fibre was the nutrient with the highest compliance, followed by proteins. On the contrary, NCs about sugars and “light/lite” had the lowest compliance (less than 40%) (Table 2). This is mainly because the NC is not accompanied by an indication of the characteristic(s) which make(s) the food ‘light’ (49 of 87 total “light/lite” NCs). The specific NCs, “very low sodium/salt” was 100% correct. Other NCs, such as “source of protein”, “low sugars”, “source of fibre”, “low fat”, “fat-free” and “source of vitamin D” were more than 90% correct (Table S3). On the contrary, none of the NCs related to saturated fats were correct (Table 3). Most NCs about omega 3 were also incorrect, mainly for two reasons: the total amount in the food was not provided or the specific amount of each of the omega 3 fatty acids was not shown (ALA, EPA, DHA) (Table 3 and Table S3).

For specific minerals, compliance was lower than 35%, except for those NCs about calcium (80%) and zinc (57.1%) (Table S3). Regarding vitamins, the compliance was heterogeneous: above 75% of all the NCs about vitamins D, B2, B12 and B3 were correct, while only 7.4% for those about vitamin B5 (Table S3). As for the comparative NCs, 53.7% were incorrect, while 43% correct and 3.3% non-evaluable.

The distribution of correct NCs was heterogeneous among food groups, as observed in Table 2. Vegetables was the group with the highest compliance rate, driven mostly by 95–100% correct NCs about fat and fibre. Non-alcoholic beverages followed, helped by a high number of correct NCs about vitamins, minerals and energy. Although the most frequent category for dairy, fat, had a high rate of correct NCs, the rest pushed down the compliance of the entire group. This was also the case for meat (fat and salt/sodium) and cereals (fibre and vitamins).

The rest of the groups presented a compliance below 50% (Table 2). A low rate of correct NCs about fat, minerals and a significant number of non-authorised were responsible for this in the legumes group. For fruits, the two main categories, sugars and minerals, along with energy, drove the low compliance. A similar situation for fish and seafood regarding fat and “naturally/natural”. In fact, 51 of 56 NCs about omega 3 fatty acids were incorrect. The lowest compliance was for nuts and seeds, mostly due to the NCs about minerals and vitamins, with more than 80% incorrect.

**Figure 2:**
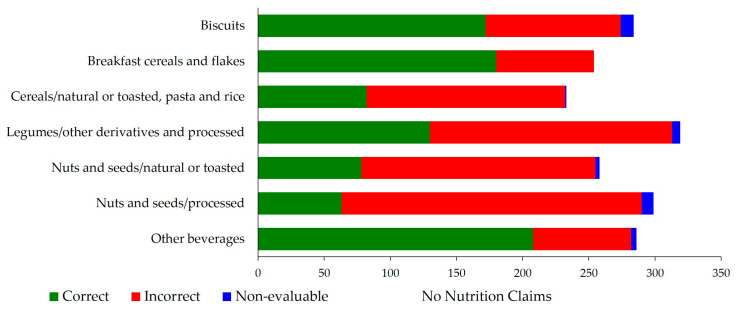
NCs compliance in main food subgroups (>200 NCs).

As for the food subgroups with more than 200 NCs (Figure 2), the highest compliance was for other beverages (72.7%), followed by breakfast cereals and flakes (70.9%). On the contrary, three of the subgroups had a 35% compliance or below (cereals/natural or toasted, pasta and rice and the two nuts and seeds subgroups) (Figure 2).

Authorised NCs failed to comply with regulation mostly for two reasons (Table 3). Failing to comply with the specific conditions only accounted for around one third of all the incorrect authorised NCs (35.4%) (Table 3). However, 59.8% were incorrect because the amount of the substance was not stated in the label (915 of 1529 NCs) (Table 3). Trans fatty acids never showed up in the label and that was the reason for many of the incorrect NCs about saturated fats (64.8%) (Table 3 and Table S3). Another major reason was failure to reach the minimum amount of the substance or to surpass the threshold established by the regulation (21.6%). The main reason for the claim “with no added sugar” to be incorrect was that the indication “contains naturally occurring sugars” was not included in the label (90 of 96 incorrect NCs).

## 4. Discussion

In this work, we systematically assess the prevalence and compliance of NCs in a large sample of products in the Spanish market. Our results show that 36.1% of foods carried NCs, at a rate of 3.3 NCs/food. The prevalence was very heterogeneous among food groups, with nuts and seeds, legumes and non-alcoholic beverages as the ones with the highest rates. Micronutrients, fat, fibre and sugars were the nutrients most frequently referred to in NCs. Overall, the compliance was low, with 49.2% correct. Fibre and proteins were the nutrients with more correct NCs, while vegetables and non-alcoholic beverages had the most correct NCS of the food groups. The main reason for incorrect NCs was because the amount of the nutrient referred to by the NC was not stated in the label.

NC classification and conditions for compliance are different depending on the rules set out by the institutions in charge. The present work followed Regulation (EC) No 1924/2006 [1]. Although not specifically stated, it is inferred from the regulation that, for EC, NCs include “nutrient content” and “comparative nutrient claims” [1]. The INFORMAS taxonomy, developed by The International Network for Food and Obesity/Non-Communicable Diseases (NCDs) Research, Monitoring and Action Support, also includes health-related ingredient claims among NCs [27]. In Canada, the term “nutrition claim” also referrers to “nutrition content claims”, “health claims” and front-of-package symbols [28]. Therefore, in this discussion we have compared our own results with those on nutrition content and comparative nutrient claims from previous publications.

### 4.1. Prevalence of Nutrition Claims

Our data show that 36.1% of foods in the BADALI database bore NCs, at a rate of 3.3 NCs/food. Three previous works carried out in Spain delivered variable results. A five-country study in 2016, with more than 2000 total foods, showed a 23% prevalence for Spain (405 foods) and a rate of 2.1 NCs/food. The average for the five countries was 21% (2.0 NCs/food) and a maximum of 29% for the UK (2.1 NCs/food) [29]. In the present work, 21 was the highest number of NCs on a single product, while it was 12 for Spain and 13 for all five countries in the same study [29]. In fact, we found 11 products with more than 12 NCs/each. A similar value was obtained in another publication evaluating 4568 foods in the Spanish market, with 20% prevalence. However, in this case, authors only considered NCs about fat, sugars, fibre and salt [30]. In fact, those nutrients only represented around 46% of all NCs in our study. Therefore, higher values would have been expected if they had considered all of them. Another small study of 88 products advertised on TV showed that 50% of them had NCs [31].

Low prevalence was obtained in the analysis of 7526 products in New Zealand. Nutrient content claims were present in 25.4% of foods, while comparative claims were in 7.8% of them (values cannot be added since foods may carry both kind of claims) [32]. The rates were lower than the ones in the present study (3.3 NCs/food here and 1.4-1.8 claims/food in Al-Ani et al. [32]). In fact, authors found 4288 NCs in 7526 total foods [32], while in our case, 3197 foods had 3839 NCs. A small study in the UK (382 foods) obtained even lower prevalence: 16.5% of foods carried nutrient content claims, while 4.5% were comparative, with a rate of 1.3 and 1.1 NCs/food respectively [33]. The analysis of 3491 foods collected in the five largest food retailers in Brazil in 2017 found NCs in 28.5% of foods [27]. A higher frequency was also shown in other studies in Australia, Canada and Ireland. The Canadian study analysed more than 15,000 samples and found that 43% of foods carried nutrition content claims [28]. The Irish work examined 1880 products and obtained 47% prevalence and a low rate of 1.8 NCs/food [34]. The Australian sample was much smaller, 215 ultra-processed foods, with 56.3% of them carrying nutrient content claims and 10.2% comparative nutrient claims [35].

NCs prevalence by food groups varied greatly in the different publications. Breakfast cereals have been extensively studied regarding NCs. Our results show that 77% of them bore NCs, which is in agreement with a previous work in Italy (70%) [36]. Another study in New Zealand, considering both breakfast cereals and cereal bars, rendered lower values (around 60%) [32]. A prevalence over 80% were obtained in several studies in Brasil, Australia and Ireland [6,27,34,35,37]. However, the rate in the present work was higher (4.3 NCs/food) than in Lalor et al. (2.2 NCs/food) [34].

The frequency of NCs in biscuits in the Brasilian study (40.4%) was similar to our data (46.3%) [27], while it was lower in the Irish work (32%) and in a 16-country work of 737 sweet biscuits and chips (around 30%) [34,38]. In fact, 1.55 NCs/food was the rate obtained in Lalor et al. [34] and 2.8 NCs/food in the present report. Our result in non-alcoholic beverages (53.1%) was the same as in two preceding studies in UK (53%) and Australia (54%) [33,37]. However, lower values were obtained in several other countries, including Spain (30–40%) [28,29,32]. Our data on legumes and vegetables were in agreement with previous publications from Canada and Ireland [28,34]. However, the rate we obtained for legumes (4.8 NCs/food) was higher than in the Irish study (three NCs/food) [34].

Our prevalence values for sauces and condiments and sweets and chocolates fell within the same range as past studies (10–20%) [27,28,29,32]. Hieke et al. obtained a similar rate (two NCs/food) as the one reported here (1.8 NCs/food) for sweets and chocolates, although Al-Ani et al. found lower rates (1.1–1.3 NCs/food) [29,32]. Similarly, our results on dairy products (31%—2.1 NCs/food) were comparable to the ones obtained in the five-country study (28%—2.3 NCs/food) and in New Zealand (23.9%—2.1 NCs/food and 14.4%—1.4 NCs/food for nutrient content and comparative claims, respectively), both for prevalence and rate [29,32]. However, other authors reported much higher values (56–62% prevalence) [28,33]. Data on fish and seafood, meat and nuts and seeds groups were quite variable in preceding works and our results added to this variability. For fish and seafood, our result (31.8%) is intermediate between the one obtained by Franco-Arellano et al. (44.8%) and Hieke et al. (25.6%), with a higher rate (2.3 NCs/food in our case; 1.5 NCs/food in Hieke et al.) [28,29]. NCs prevalence in meat in our study was higher (27.9%) than the one in the three previous reports (9.5–16%), while lower than in the Canadian work (34.4%) [27,28,29,34]. However, our rate (1.6 NCs/food) was lower than in the five-country study (2.3 NCs/food) [29]. We report very high NCs prevalence for nuts & seeds (63.2%), which is higher than in previous articles (less than 15 to 53%) [27,28].

NCs prevalence in fermented milk subgroup was lower in the present work (35%) than in Lalor et al. (55%), but with similar rates (1.6 and 1.7 NCs/food respectively) [34]. Regarding cheese, although only 28% had NCs in our study compared to 41% in the Irish work, higher rate was observed (2.2 NCs/food here, 1.6 NCs/food in Lalor et al.) [34]. Our results in juices and canned vegetables are strikingly different to previous reports. Prevalence was much lower for the first food group, while as much as 10-fold higher than others for the second [6,27,34]. Fifty-one percent was the value for fruit beverages and soft drinks here, which is similar to the Irish study (45%), although with a double rate [34]. Other beverages presented a slight increase in the present report (72%) compared to the Brasilian one (around 65%) [27]. As for cereal bars, Hughes et al. found 77% prevalence, while we obtained 65% [37].

Regarding nutrients, micronutrients, fat, fibre and sugars were the most frequently claimed in the present analysis, which is in agreement with previous studies [29,33,34,39,40].

As mentioned above, the differences among studies may be due to divergent definitions of NCs. The food sample analysed may also be an important factor because heterogeneity may be smaller in those publications focused on only 1–2 food groups than in general ones. In addition, some works used quite a low number of foods and the results may depend much on the type of foods selected. This is supported by our own data showing a very different prevalence depending on the groups and subgroups analysed. Even in those works using more than 3000 foods, including ours, they may not be representative given the huge amount present in the market.

Another important factor influencing variability may be the different criteria used to group foods. In fact, some works do not describe the food groups. The date when food information was collected may also affect the results. The papers reviewed in the Discussion contained data collected from 2000 to 2019. Since the use of NCs is a quite recent activity, regulations issued along such a wide range of years may greatly affect the way NCs are used.

### 4.2. Nutrition Claims Compliance

Only a few publications address the compliance of NCs. The European regulation is quite clear in the wording and conditions for NCs. However, this is not the case for the Australian–New Zealand standard, which does not prescribe wording for claims. As a consequence, the use of these NCs is subject to interpretation by anyone involved in the process, including authors evaluating them [6]. Therefore, we should be cautious when comparing results.

Since the use of NCs in Spain is regulated by European Regulation (EC) No 1924/2006 and compliance is mandatory, the full correctness of NCs should be guaranteed [1]. However, our results show otherwise. Surprisingly, less than half of the NCs analysed comply with the regulation, which is much lower than the rate obtained previously in a small Spanish sample (88 foods, 89% correct NCs) [31]. Similar results were obtained for breakfast cereals in the Australian market with 94% compliance for nutrient content claims [6]. On the contrary, the analysis of 215 ultra-processed foods in Australia showed a very low compliance: 18.6% of products had entirely correct nutrient content claims and 27.3% for comparative nutrient claims [35]. However, these data cannot be compared with our results because they use products instead of NCs and also because the conditions for correctness are very restrictive (only products with all NCs correct were considered in the 18.6% value).

The main reason for incorrect NCs in the present work was that the amount of the substance claimed was not stated in the label, which is against Article 7 of the Regulation (EC) No 1924/2006 [1]. Failure to comply with the specific conditions of NCs was another important reason. This proves a very low knowledge of the food industry as to the use of this regulation. It is also a proof of the failure of the authorities to enforce the mandatory regulation.

### 4.3. Nutrition Quality of Foods Bearing Nutrition Claims

A priori, one may expect that NCs were only used in healthy foods, so not to mislead consumers. Some analyses have shown an overall better nutrient composition for critical nutrients and energy among foods with NCs compared to those without, although with some conflicting results [27,28,33,36,41,42,43,44]. However, the question still remains as to whether foods carrying NCs are healthy. Studies show that a large proportion of foods bearing NCs were considered “less healthy” or high in critical nutrients when nutrient profile models were applied [27,28,32].

Some of the groups and subgroups analysed in the present work are considered unhealthy or less healthy according to the Nutrient Profile Model developed by the WHO Regional Office for Europe [45]. This is the case for biscuits, which has a large proportion of NCs, with 36% incorrect. Sweets and chocolate are also considered less healthy and they display a significant number of NCs, with 60% incorrect. For processed meat (fresh meat was not included in the database) the rate was more than 40%. Processed meat was classified as group 1, carcinogenic to humans by World Health Organization in 2015 [46].

Therefore, carrying NCs is not a guarantee of healthy food and this may mislead consumers. In fact, nutrition and health claims may provide a “health halo” to the product bearing them so that they are considered healthier than they actually are [5,6,7,8].

### 4.4. Strengths and Limitations

The strengths of this work are several:

- More than 3000 foods were analysed, which is in the medium range of the publications on this subject;
- Foods from all groups were analysed, which provided an overview of products in the Spanish market;
- Data were collected over a several year period (2013–2019), several years after Regulation (EC) No 1924/2006 was in force;
- Most foods included in the database were processed. Processed foods were the ones generally carrying NCs, while fresh foods do not usually make use of them;
- Only a few publications analyse the compliance of NCs according to the European regulation.

Our work has a few important limitations:

- Data collected were reliant on the accuracy of the information provided on the manufacturer’s webpage;
- Some NCs in the packages may be missing because of the low resolution of some of the images in the database;
- Selection of brands did not follow criteria based on customer’s purchase or the most popular products;
- The 3197 foods analysed may not be representative due to the huge amount of foods available in the market;
- When evaluating NCs by food group and subgroup, the data are limited compared to those studies focused on one type of foods;
- There may have been changes in NCs and the nutrient composition for some foods since information was collected.

## 5. Conclusions

The results of our study reveal that the aim of the EC to “ensure a high level of protection for consumers and to facilitate their choice” regarding NCs has not been fulfilled [1]. A high number of NCs were used, many on unhealthy foods, and in spite of being mandatory, half of them were incorrect according to Regulation (EC) No 1924/2006 [1]. Therefore, customers may require extra information regarding the correctness of NCs in order to make healthy choices. This is one of the aims of the BADALI project, freely available online [19]. The foods examined in this study can be consulted in the BADALI webpage and the analysis of the NCs made on every food is included. As an educational tool, fresh foods that may potentially carry the same NCs are suggested as alternatives [47].

This situation is particularly worrying because of the effect of NCs on customers’ behaviour and perceptions. Therefore, instead of being used to promote a healthy diet, in practice, NCs become an additional tool for manufacturers to improve their sales. A system should be implemented to guarantee the proper use of NCs. A real commitment by the authorities is required in order to truly promote a healthy diet through this and other food labelling techniques.

## Figures and Tables

**Table 1:** Number of foods and nutrition claims (NCs) by group.

Food Groups	No Foods (% of Total Products Surveyed)	No Foods with NCs (% of Total Products with NCs)	No NCs/Food ^2^
Total	3197 (100)	1155 (36.1) ^1^	3.3
Cereals	796 (24.9)	329 (28.5)	3.2
Dairy	440 (13.8)	136 (11.8)	2.1
Fats	13 (0.4)	11 (1.0)	3.6
Fish and seafood	311 (9.7)	99 (8.6)	2.3
Fruits	172 (5.4)	42 (3.6)	2.6
Legumes	254 (7.9)	138 (11.9)	4.8
Meat	262 (8.2)	73 (6.3)	1.6
Non-alcoholic beverages	260 (8.1)	138 (11.9)	3.6
Nuts and seeds	95 (3.0)	60 (5.2)	9.3
Sauces and condiments	95 (3.0)	14 (1.2)	1.4
Sweets and chocolates	270 (8.4)	39 (3.4)	1.8
Vegetables	229 (7.2)	76 (6.6)	2.4

^1^ % of total products surveyed; ^2^ Calculated as: Total or group No NCs/total or group No foods with NC.

**Table 2:** Number of NCs and compliance by group and nutrient.

Food Group and Compliance	Total (%)	Energy (%)	Fat (%)	Fibre (%)	Light/Lite (%)	Minerals (%)	Naturally/Natural (%)	Proteins (%)	Reduced (others) (%)	Salt/Sodium (%)	Sugars (%)	Vitamins (%)	Non-Authorised (%)
Total	3839 (100) ^1^	90 (2.3) ^1^	692 (18) ^1^	454 (11.8) ^1^	87 (2.3) ^1^	488 (12.7) ^1^	84 (2.2) ^1^	281 (7.3) ^1^	4 (0.1) ^1^	243 (6.3) ^1^	392 (10.2) ^1^	669 (17.4) ^1^	355 (9.4) ^1^
Correct	1890 (49.2) ^1^	56 (62.2)	306 (44.2)	399 (87.9)	32 (36.8)	202 (41.4)	45 (53.6)	203 (72.2)	0 (0)	144 (59.3)	147 (37.5)	356 (53.2)	0 (0)
Incorrect	1884 (49.1) ^1^	33 (36.7)	383 (55.3)	46 (10.1)	54 (62.1)	275 (56.4)	36 (42.9)	68 (24.2)	4 (100)	90 (37)	227 (57.9)	313 (46.8)	355 (100)
Non-evaluable	65 (1.7) ^1^	1 (1.1)	3 (0.4)	9 (2)	1 (1.1)	11 (2.3)	3 (3.6)	10 (3.6)	0 (0)	9 (3.7)	18 (4.6)	0 (0)	-- ^2^
Cereals	1058 (27.6) ^1^	1	130	215	13	100	9	45	1	75	159	225	85
Correct	546 (51.6)	0 (0)	49 (37.7)	186 (86.5)	0 (0)	47 (47)	8 (88.9)	26 (57.8)	0 (0)	37 (49.3)	46 (28.9)	147 (65.3)	0 (0)
Incorrect	498 (47.1)	1 (100)	81 (62.3)	26 (12.1)	13 (100)	53 (53)	1 (11.1)	19 (42.2)	1 (100)	37 (49.3)	103 (64.8)	78 (34.7)	85 (100)
Non-evaluable	14 (1.3)	0 (0)	0 (0)	3 (1.4)	0 (0)	0 (0)	0 (0)	0 (0)	0 (0)	1 (1.3)	10 (6.3)	0 (0)	-- ^2^
Dairy	288 (7.5) ^1^	6	69	4	28	50	5	21	0	10	7	47	41
Correct	171 (59.4)	5 (83.3)	53 (76.8)	4 (100)	15 (53.6)	33 (66)	3 (60)	21 (100)	0 (0)	4 (40)	0 (0)	33 (70.2)	0 (0)
Incorrect	113 (39.2)	1 (16.7)	16 (23.2)	0 (0)	13 (46.4)	17 (34)	0 (0)	0 (0)	0 (0)	4 (40)	7 (100)	14 (29.8)	41 (100)
Non-evaluable	4 (1.4)	0 (0)	0 (0)	0 (0)	0 (0)	0 (0)	2 (40)	0 (0)	0 (0)	2 (20)	0 (0)	0 (0)	-- ^2^
Fish and seafood	230 (6.0) ^1^	8	88	0	9	7	45	27	0	13	0	3	30
Correct	80 (34.8)	0 (0)	20 (22.7)	0 (0)	3 (33.3)	4 (57.1)	17 (37.8)	26 (96.3)	0 (0)	10 (76.9)	0 (0)	0 (0)	0 (0)
Incorrect	145 (63)	7 (87.5)	68 (77.3)	0 (0)	5 (55.6)	3 (42.9)	27 (60)	0 (0)	0 (0)	2 (15.4)	0 (0)	3 (100)	30 (100)
Non-evaluable	5 (2.2)	1 (12.5)	00 (0)	0 (0)	1 (11.1)	0 (0)	1 (2.2)	1 (3.7)	0 (0)	1 (7.7)	0 (0)	0 (0)	-- ^2^
Fruits	111 (2.9) ^1^	18	0	19	7	21	0	1	0	0	31	12	2
Correct	48 (43.2)	4 (22.2)	0 (0)	18 (94.7)	4 (57.1)	8 (38.1)	0 (0)	1 (100)	0 (0)	0 (0)	11 (35.5)	2 (16.7)	0 (0)
Incorrect	63 (56.8)	14 (77.8)	0 (0)	1 (5.3)	3 (42.9)	13 (61.9)	0 (0)	0 (0)	0 (0)	0 (0)	20 (64.5)	10 (83.3)	2 (100)
Non-evaluable	0 (0)	0 (0)	0 (0)	0 (0)	0 (0)	0 (0)	0 (0)	0 (0)	0 (0)	0 (0)	0 (0)	0 (0)	-- ^2^
Legumes	666 (17.3) ^1^	0	166	78	2	85	20	94	1	45	34	63	78
Correct	307 (46.1)	0 (0)	53 (31.9)	62 (79.5)	0 (0)	37 (43.5)	16 (80)	68 (72.3)	0 (0)	26 (57.8)	18 (52.9)	27 (42.9)	0 (0)
Incorrect	339 (50.9)	0 (0)	110 (66.3)	11 (14.1)	2 (100)	39 (45.9)	4 (20)	23 (24.5)	1 (100)	19 (42.2)	16 (47.1)	36 (57.1)	78 (100)
Non-evaluable	20 (3)	0 (0)	3 (1.8)	5 (6.4)	0 (0)	9 (10.6)	0 (0)	3 (3.2)	0 (0)	0 (0)	0 (0)	0 (0)	-- ^2^
Meat	120 (3.1) ^1^	0	61	0	0	14	0	15	2	17	0	6	5
Correct	70 (58.3)	0 (0)	43 (70.5)	0 (0)	0 (0)	0 (0)	0 (0)	15 (100)	0 (0)	12 (70.6)	0 (0)	0 (0)	0 (0)
Incorrect	50 (41.7)	0 (0)	18 (29.5)	0 (0)	0 (0)	14 (100)	0 (0)	0 (0)	2 (100)	5 (29.4)	0 (0)	6 (100)	5 (100)
Non-evaluable	0 (0)	0 (0)	0 (0)	0 (0)	0 (0)	0 (0)	0 (0)	0 (0)	0 (0)	0 (0)	0 (0)	0 (0)	-- ^2^
Non-alcoholic beverages	501 (13.1) ^1^	43	82	13	15	49	0	26	0	30	61	157	25
Correct	337 (67.3)	40 (93)	38 (46.3)	12 (92.3)	9 (60)	41 (83.7)	0 (0)	26 (100)	0 (0)	30 (100)	28 (45.9)	113 (72)	0 (0)
Incorrect	160 (31.9)	3 (7)	44 (53.7)	1 (7.7)	6 (40)	8 (16.3)	0 (0)	0 (0)	0 (0)	0 (0)	29 (47.5)	44 (28)	25 (100)
Non-evaluable	4 (0.8)	0 (0)	0 (0)	0 (0)	0 (0)	0 (0)	0 (0)	0 (0)	0 (0)	0 (0)	4 (6.6)	0 (0)	-- ^2^
Nuts & seeds	557 (14.5) ^1^	3	35	66	0	156	4	47	0	36	23	130	57
Correct	141 (25.3)	0 (0)	2 (5.7)	61 (92.4)	0 (0)	28 (17.9)	1 (25)	16 (34)	0 (0)	14 (38.9)	4 (17.4)	15 (11.5)	0 (0)
Incorrect	404 (72.5)	3 (100)	33 (94.3)	4 (6.1)	0 (0)	126 (80.8)	3 (75)	25 (53.2)	0 (0)	19 (52.8)	19 (82.6)	115 (88.5)	57 (100)
Non-evaluable	12 (2.2)	0 (0)	0 (0)	1 (1.5)	0 (0)	2 (1.3)	0 (0)	6 (12.8)	0 (0)	3 (8.3)	0 (0)	0 (0)	-- ^2^
Sweets and chocolates	70 (1.8) ^1^	1	8	3	2	4	0	0	0	2	42	3	5
Correct	24 (34.3)	0 (0)	0 (0)	2 (66.7)	0 (0)	2 (50)	0 (0)	0 (0)	0 (0)	0 (0)	20 (47.6)	0 (0)	0 (0)
Incorrect	42 (60)	1 (100)	8 (100)	1 (33.3)	2 (100)	2 (50)	0 (0)	0 (0)	0 (0)	1 (50)	19 (45.2)	3 (100)	5 (100)
Non-evaluable	4 (5.7)	0 (0)	0 (0)	0 (0)	0 (0)	0 (0)	0 (0)	0 (0)	0 (0)	1 (50)	3 (7.1)	0 (0)	-- ^2^
Vegetables	179 (4.7) ^1^	8	37	55	6	0	1	5	0	15	28	8	16
Correct	134 (74.9)	6 (75)	37 (100)	53 (96.4)	0 (0)	0 (0)	0 (0)	4 (80)	0 (0)	11 (73.3)	16 (57.1)	7 (87.5)	0 (0)
Incorrect	43 (24)	2 (25)	0 (0)	2 (3.6)	6 (100)	0 (0)	1 (100)	1 (20)	0 (0)	3 (20)	11 (39.3)	1 (12.5)	16 (100)
Non-evaluable	2 (1.1)	0	0	0	0	0	0	0	0	1 (6.7)	1 (3.6)	0	-- ^2^

^1^ % of the total NCs; ^2^ “Non-evaluable” only applies to authorised NCs.

**Table 3:** Reasons for failing to comply with the regulation (only authorised NCs).

Reasons	No NCs
Amount of substance not stated	
Amount of specific omega 3 fatty acids not provided	34
Amount of other substances not stated	743
Amount of trans-fatty acids not stated	138
Failure to comply with specific conditions	
Comparative—not meeting condition	73
“Contains naturally occurring sugars” not stated	90
Failure to comply with minimum or maximum stablished	330
No indication of the characteristic which makes the food light	49
Other reasons	
Naturally/Natural—incorrect associated NC	36
Other reasons	36

## Supplementary Materials

The following are available online at https://www.mdpi.com/2072-6643/12/10/2943/s1, Table S1: Description of food groups and subgroups, Table S2: Foods with NCs and number of NCs by subgroup, Table S3: List of all NCs authorized with total frequency, correct, incorrect and non-evaluable, Table S4: Non-authorised claims used in foods, Figure S1: Distribution of foods according to the number of NCs.
