# Supplementary material for: Nutrition Claims Frequency and Compliance in a Food Sample of the Spanish Market: The BADALI Study
Source: Nutrients. 2020 Sep 25;12(10):2943. doi: 10.3390/nu12102943 (PMC7599968; doi:10.3390/nu12102943)
Supplement: Supplementary file 1 [file nutrients-12-02943-s001.pdf]

Supplementary Materials

Table S1. Description of food groups and subgroups

Food groups	Food subgroups	Foods included
**Cereals**	Biscuits	Filled, regular, with chocolate, polialcohols.
	Bran, flour and germ	Bran, germ and flours (refined and whole-grain)
	Bread (also sliced)	Bread (normal, sliced, hamburger, doggy), refined, whole-grain, with seeds.
	Breakfast cereals and flakes	Puffed rice, oatmeal, flakes.
	Cakes and pastries	Donuts, muffins, gluten-free cupcake, croissants, profiteroles, ready-to-use mixes for pastries
	Cereal bars	Plain, with chocolate, nuts, (dry) fruits, polialcohols
	Cereal cakes and toasts	Cakes (rice, wheat, corn, oat, spelt), wheat toasts.
	Natural or toasted, pasta and rice	Amaranth, quinoa, oats, barley, rye, millet, wheat, corn, spelt. Refined and whole-grain pasta and rice (brown).
	Other derivatives and processed	Snacks, pizza base, doughs, nachos, wheat sticks, dehydrated soup, pancake mix, breadcrumbs.
**Dairy**	Cheese	Fresh, cured or semi-cured manchego, melted, with spices; cow, sheep, goat.
	Dairy desserts	Custard, flan, catalane cream, chocolate and other flavoured mousses, rice pudding
	Derivatives	Butter, cream, cheese mousse, spreading creams with cheese.
	Fermented milk	Yogurts, other fermented milk
	Milk-dairy beverages	Milk (liquid, powder, condensed or evaporated), milk shakes, milk with other ingredients
**Fats**	--	Lard, margarines
**Fish and seafood**	Derivatives	Tuna and anchovie pate, surimi.
	Processed	Canned, smoked, marinated. Roe, liver. Tuna, sardines, prawns, mackerel, salmon, octopus, mussels and other seafood.
	Whole or chopped	Fresh, chilled or frozen with no added ingredients.
**Fruits**	Derivatives	Olives, olive pates, canned fruit, candied fruit, jams, fruit spreads, sweet quinces.
	Dry	Figs, dates, plums, raisins.

Table S1 (cont). Description of food groups and subgroups

Food groups	Food subgroups	Foods included
**Legumes**	Canned	Beans, chickpeas, lentils, peas, green beans, soy
	Dry	Beans, chickpeas, lentils, peas, green beans, soy
	Flour and pasta-like	Chickpea, lentil, pea, carob and soy flour; pea, lentil and chickpea pasta.
	Other derivatives and processed	Fermented soy, soy desserts, chips, hummus
**Meat**	--	Cold meat, luncheon meat, pate.
**Non-alcoholic beverages**	Fruit beverages and soft drinks	Nectars, fruit drinks, musts, horchatas, soft drinks (cola, orange and lemon sodas), flavoured water with sweeteners, tonics.
	Juices	Apple, grape, tomato, peach, orange, pineapple, berries.
	Other beverages	Non-alcoholic beers, vegetable beverages (soy, oat, rice, coconut, almond)
**Nuts and seeds**	Natural or toasted	Unprocessed or toasted nuts and seeds (pumpkin, poppies, sunflower, flax, chia, hemp)
	Processed	Salted nuts
**Sauces and condiments**	Condiments and vinegar	Seasonings, vinegars.
	Sauces	Ketchup, mayonnaises, other sauces.
**Sweets and chocolates**	Chocolates	Chocolate bars, filled chocolates, chocolate powder, chocolate-coated cereal bars, ready-to-use mixes.
	Sweets	Chewing gums, caramels, wafers, honey, candies, syrups, sweet creams.
**Vegetables**	Canned	Salads, sweet corn, all kind of vegetables.
	Other derivatives and processed	Creams and soups, pickled gherkins, gazpacho, tomato-based sauces, vegetable jams.
	Tubers and derivatives	Potato chips, processed potatoes.

Table S2. Foods with NCs and number of NCs by subgroup

Food groups/subgroups	No foods	No foods with NCs	% Foods with NCs within the subgroup	No NCs	No NCs/food [1]
**Cereals**					
Biscuits	218	101	46.3	284	2.8
Bran, flour and germ	50	27	54.0	83	3.1
Bread (also sandwich)	29	8	27.6	8	1.0
Breakfast cereals and flakes	77	59	76.6	254	4.3
Cakes and pastries	75	5	6.7	14	2.8
Cereal bars	26	17	65.4	36	2.1
Cereal cakes and toasts	54	19	35.2	22	1.2
Natural or toasted, pasta and rice	163	52	31.9	233	4.5
Other derivatives and processed	104	41	39.4	124	3.0
**Dairy**					
Cheese	164	46	28	99	2.2
Dairy desserts	32	2	6.3	5	2.5
Derivatives	63	14	22.2	23	1.6
Fermented milk	121	42	34.7	68	1.6
Milk-dairy beverages	60	32	53.3	93	3.6
**Fish and seafood**					
Derivatives	22	12	54.5	24	2.0
Processed	234	74	31.6	178	2.4
Whole or chopped	55	13	23.6	28	2.2
**Fruits**					
Derivatives	155	33	21.3	69	2.1
Dry	17	9	52.9	42	4.7
**Legumes**					
Canned	53	37	69.8	119	3.2
Dry	123	32	26.0	173	5.4
Flour and pasta-like	15	15	100	55	3.7
Other derivatives and processed	63	54	85.7	319	5.9
**Non-alcoholic beverages**					
Fruit beverages and soft drinks	156	79	50.6	194	2.5
Juices	36	10	27.8	21	2.1
Other beverages	68	49	72.1	286	5.8
**Nuts and seeds**					
Natural or toasted	45	30	66.7	258	8.6
Processed	50	30	60.0	299	10
**Sauces and condiments**					
Condiments and vinegars	11	0	0.0	0	0.0
Sauces	84	14	16.7	19	1.4
**Sweets and chocolates**					
Chocolates	231	33	14.3	60	1.8
Sweets	39	6	15.4	10	1.7
**Vegetables**					
Canned	81	46	56.8	123	2.7
Other derivatives and processed	103	19	18.4	27	1.4
Tubers and derivatives	45	11	24.4	29	2.6

[1]Only considered foods with NCs

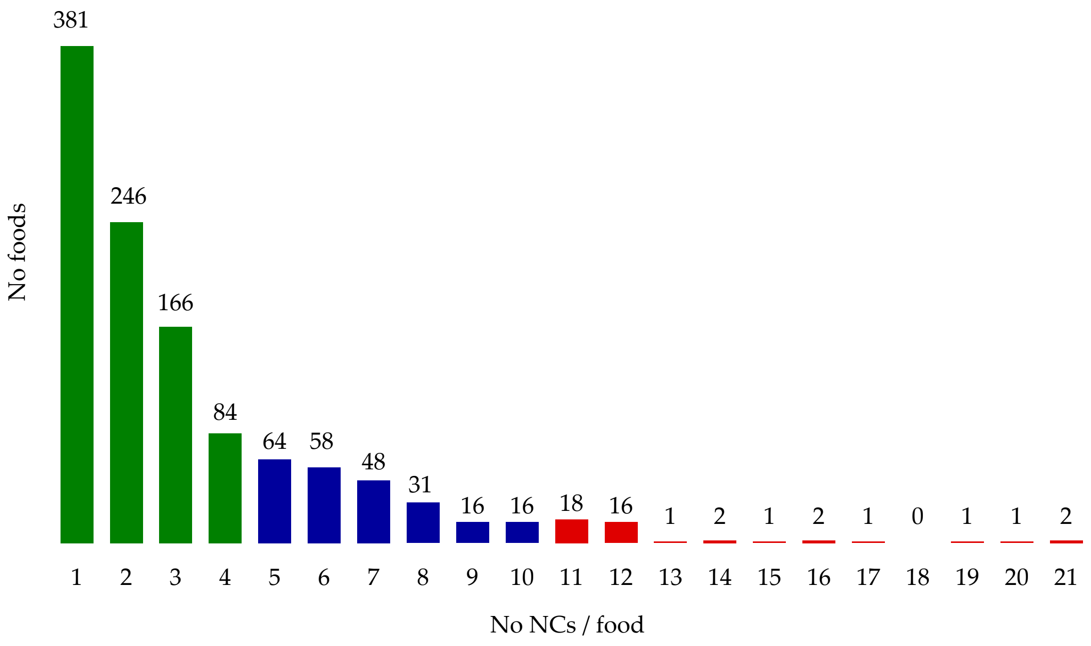

Figure S1. Distribution of foods according to the number of NCs

Table S3. List of all NCs authorized with total frequency, correct, incorrect and non-evaluable

Specific NC	Total	Correct		Incorrect		Non-evaluable	
	n	n	%	n	%	n	%
Energy-free	22	12	54.5	10	45.5	0	0
Energy-reduced	35	27	77.1	7	20	1	2.9
Fat-free	98	89	90.8	9	9.2	0	0
High calcium	31	17	54.8	13	41.9	1	3.2
High fibre	214	186	86.9	24	11.2	4	1.9
High iron	33	13	39.4	14	48.5	4	12.1
High magnesium	48	5	10.4	42	87.5	1	2.1
High minerals (general)	10	0	0	10	100	0	0
High omega-3 fatty acids	42	0	0	42	100	0	0
High phosphorus	46	4	8.7	42	91.3	0	0
High polyunsaturated fat	9	4	44.4	5	55.6	0	0
High potassium	8	4	50	4	50	0	0
High protein	146	79	54.1	58	39.7	9	6.2
High selenium	1	0	0	1	100	0	0
High unsaturated fat	46	22	47.8	21	45.7	3	6.5
High vitamin A	8	0	0	8	100	0	0
High vitamin B1	15	9	60	6	40	0	0
High vitamin B12	11	8	72.7	3	27.3	0	0
High vitamin B2	9	8	88.9	1	11.1	0	0
High vitamin B3	10	8	80	2	20	0	0
High vitamin B6	12	8	66.7	4	33.3	0	0
High vitamin B9	12	9	75	3	25	0	0
High vitamin C	16	7	43.75	9	56.25	0	0
High vitamin D	6	4	66.7	2	33.3	0	0
High vitamin E	16	9	56.3	7	43.8	0	0
High vitamin K	2	0	0	2	100	0	0
High vitamins (general)	12	0	0	12	100	0	0
High vitamins B	7	0	0	7	100	0	0
High zinc	2	0	0	2	100	0	0
Increased fibre	6	2	33.3	1	16.7	3	50
Increased minerals (general and specific)	9	0	0	7	77.8	2	22.2
Increased omega-3 fatty acids	5	1	20	4	80	0	0
Increased protein	6	2	33.3	4	66.7	0	0
Increased vitamins (general and specific)	2	0	0	2	100	0	0
Light	87	32	36.8	54	62.1	1	1.1
Low energy	33	17	51.5	16	48.5	0	0
Low fat	174	145	93.3	29	16.7	0	0
Low saturated fat	164	0	0	164	100	0	0
Low sodium/salt	39	31	79.5	8	20.5	0	0
Low sugars	35	32	91.4	3	8.6	0	0
Naturally / Natural	84	45	53.6	36	42.9	3	3.6
No added sodium/salt	21	15	71.4	1	4.8	5	23.8
Reduced (others)	4	0	0	4	100	0	0
Reduced fat	42	31	73.8	11	26.2	0	0
Reduced fibre	1	0	0	1	100	0	0
Reduced protein	1	0	0	1	100	0	0
Reduced saturated fat	31	0	0	31	100	0	0

Table S3 (cont). List of all NCs authorized with total frequency, correct, incorrect and non-evaluable

Specific NC	Total	Correct		Incorrect		Non-evaluable	
	n	n	%	n	%	n	%
Reduced sodium/salt	69	34	49.3	32	46.4	3	4.3
Reduced sugars	9	3	33.3	6	66.7	0	0
Saturated fat-free	18	0	0	18	100	0	0
Sodium/salt-free	72	22	30.6	49	68.1	1	1.4
Source of (number) vitamins	49	0	0	49	100	0	0
Source of boron	1	0	0	1	100	0	0
Source of calcium	124	107	86.3	16	12.9	1	0.8
Source of copper	3	0	0	3	100	0	0
Source of fibre	233	211	90.6	20	8.6	2	0.9
Source of iodine	1	0	0	1	100	0	0
Source of iron	78	24	30.8	53	67.9	1	1.3
Source of magnesium	23	11	47.8	11	47.8	1	4.3
Source of manganese	1	0	0	1	100	0	0
Source of minerals (general)	7	0	0	7	100	0	0
Source of omega-3 fatty acids	63	14	22.2	49	77.8	0	0
Source of phosphorus	31	5	16.1	26	83.1	0	0
Source of potassium	14	2	14.3	12	85.7	0	0
Source of protein	128	122	95.3	5	3.9	1	0.8
Source of selenium	5	2	40	3	60	0	0
Source of vitamin A	41	23	56.1	18	43.9	0	0
Source of vitamin B1	17	12	70.6	5	29.4	0	0
Source of vitamin B12	47	40	85.1	7	14.9	0	0
Source of vitamin B2	30	25	83.3	5	16.7	0	0
Source of vitamin B3	18	14	77.8	4	22.2	0	0
Source of vitamin B5	27	2	7.4	25	92.6	0	0
Source of vitamin B6	59	33	55.9	26	44.1	0	0
Source of vitamin B8	1	1	100	0	0	0	0
Source of vitamin B9	58	28	48.3	30	51.7	0	0
Source of vitamin C	31	17	54.8	14	45.2	0	0
Source of vitamin D	65	59	90.8	6	9.2	0	0
Source of vitamin E	65	26	40	38	60	0	0
Source of vitamin K	2	0	0	2	100	0	0
Source of vitamins (general)	16	6	37.5	10	62.5	0	0
Source of vitamins B	5	0	0	5	100	0	0
Source of zinc	12	8	66.7	4	33.3	0	0
Sugars free	207	82	39.6	122	58.9	3	1.4
Very low sodium/salt	42	42	100	0	0	0	0
With no added sugars	141	30	21.3	96	68.1	15	10.6

Table S4. Non-authorised claims used in foods

Type	No
**General**	**227**
Content of nutrients	12
Nutritive	70
Others	46
**Specific nutrients**	**127**
Aminoacids	6
Carbohydrates	41
Cholesterol	6
Energy	10
Fiber	2
Lipids	111
Other components	13
Others	3
Proteins	32
Vitamins and minerals	3
